# Factors of Seeking Professional Psychological Help by the Bereaved by Suicide

**DOI:** 10.3389/fpsyg.2020.00592

**Published:** 2020-04-08

**Authors:** Odeta Geležėlytė, Danutė Gailienė, Jolanta Latakienė, Eglė Mažulytė-Rašytinė, Paulius Skruibis, Said Dadašev, Dovilė Grigienė

**Affiliations:** Suicide Research Centre, Institute of Psychology, Vilnius University, Vilnius, Lithuania

**Keywords:** suicide, bereavement, help-seeking, professional help, stigmatization, guilt

## Abstract

**Background:**

Studies show that people bereaved by suicide often feel a strong need for professional help. It is hypothesized that aspects related to suicide bereavement, such as stigmatization, shame or guilt, hinder help-seeking process of the bereaved. However, little is known about help-seeking behaviors of people who has lost someone due to suicide.

**Aims:**

This study was conducted to attain a better understanding of the contributing factors, including the specific features of grief following suicide, to help-seeking behaviors of the bereaved by suicide.

**Methods:**

The sample consisted of 82 adults bereaved by suicide (64 female; average age 37.79, *SD* = 14.33). Instruments assessing stigmatization, shame, guilt levels, well-being, tendency to disclose emotional distress and attitudes toward seeking professional psychological help were used. The participants were also asked an open-ended question what professional help-seeking barriers they had encountered. Comparisons between the groups, logistic regression analysis and thematic analysis of the qualitative data were performed.

**Results:**

The findings revealed that bereaved participants who sought professional psychological help reported experiencing stigmatization and feeling guilty after the loss significantly more often. Also the results showed that attitudes toward mental health specialists had the highest prognostic value in predicting help-seeking behaviors of the bereaved. The participants themselves identified the gaps in the health care system as main barriers to seeking help.

**Conclusion:**

The results challenge previously spread notion that stigmatization, guilt and shame after suicide can act only as help-seeking barriers.

## Introduction

Studies show that although part of grief reactions might be seen as similar among various types of losses, the levels of perceived stigmatization, shame and guilt are usually higher among the bereaved by suicide (Harwood et al., 2002; Pitman et al., 2016). Until the 19th century, suicide was treated as a crime as well as a sin and the suicide bereaved had to suffer not only the loss of a loved one but also some ways of punishment for the suicide (Knieper, 1999). A lot has changed since then, but at least subtle ways of stigmatization, such as avoidance or rejection of the suicide bereaved, still exist (Cvinar, 2005; Hanschmidt et al., 2016). It has been shown that stigmatization contributes to mourning problems after suicide (Feigelman et al., 2009) and affect suicide-bereaved individuals’ ability to express their grief sincerely and openly (Chapple et al., 2015). Frost (2011) explains that the processes of stigmatization might vary from distant to the self to internalized ones referring to the application of negative meanings of stigma to a self-concept. Internalized stigma can be operationalized as feelings of shame (Hanschmidt et al., 2016) that are often related to defensiveness, self-hatred (Fisher and Exline, 2010), experiences of inadequacy or unworthiness (Wiklander et al., 2003) and mental distress (Bos et al., 2013). Such experiences motivate avoidance behaviors such as impulses to hide, flee or conceal oneself (Wiklander et al., 2003). Guilt might be defined as “a remorseful emotional reaction in grieving, with the recognition of having failed to live up to one’s own inner standards and expectations in relationship to the deceased and/or the death” (Li et al., 2014, p. 166). Self-blaming thoughts and feelings that the bereaved should have done something to prevent the death are common among those who experienced suicide (Tal et al., 2017). According to Clark (2012), guilt can have negative psychological consequences, when it is too much for a person to bear. Excessive or chronic feelings of guilt can also lead to acts of self-punishment (Fisher and Exline, 2010).

As people bereaved by suicide might face many difficulties, they are often in need of help and support from their social network or from professionals (Provini et al., 2000; Dyregrov, 2011). Some studies found that even up to 95% of the suicide bereaved are in need of professional help in managing their grief (Wilson and Marshall, 2010). It was found that the bereaved by suicide might be in greater need for professional help than relatives of natural deaths (De Groot et al., 2006). Receiving appropriate professional support, if it is needed, diminishes the risk of experiencing negative consequences such as emotions of sorrow, lack of energy and abandonment (Schneider et al., 2011).

However, there are some discrepancies between suicide bereaved people needing and receiving help. The main problem arises when those who are in need of professional help do not seek it. For example, in Wilson and Marshall’s (2010) study discussed before, only 44% of the suicide bereaved received professional help. Among mentioned barriers to seeking support are self-reliance, distrust of professionals, fear of being judged, reluctance to ask for help, concern for what others would think, lack of available information, poor mental health literacy, thinking no one could help or time, cost, distance (Provini et al., 2000; McMenamy et al., 2008; Wilson and Marshall, 2010; Andriessen et al., 2018).

Poor psychological condition is one of the most motivating factors to approach a professional. On the other hand, poor health, lack of energy are also mentioned among help-seeking barriers. In the study of McMenamy et al. (2008) depressed mood and lack of energy were identified as important obstacles to seeking help. Meanwhile, Wilson and Marshall (2010) found that the bereaved who were most in need of help received it. So the question if those bereaved who are most in need of help approach it remains open. It has also been claimed that more favorable attitudes toward mental health professionals are positively related to intention to seek help and to actual help-seeking behavior (Fischer and Farina, 1995; Elhai et al., 2008; Picco et al., 2016). However, we know very little about what attitudes people bereaved by suicide hold toward seeking professional help (Drapeau et al., 2015). Another factor often associated with the pursuit of psychological support is the tendency of a person to reveal personally relevant information related to distress (Kahn and Hessling, 2001; Kahn et al., 2012). Studies confirm the significance of the tendency to disclose distress to the attitudes and intentions to seek professional psychological counseling (Vogel and Wester, 2003; Nam et al., 2013). Research reveals that opening up emotions related to complicated bereavement can help to unblock the grief process (Wagner et al., 2006) and a tendency to openly discuss the death accounts for less grief difficulties (Feigelman et al., 2018). Sharing intimate information can be beneficial for growth processes following a traumatic event of suicide in the family (Levi-Belz, 2016).

It is often hypothesized that aspect related to suicide bereavement also hinder help-seeking process. Authors suggest that social stigma or shame might become serious help-seeking barriers of suicide-bereaved individuals and people after suicide attempt (Wiklander et al., 2003; Reynders et al., 2014; Skruibis et al., 2015). In their meta-analysis Hanschmidt et al. (2016) claim that stigma might lead survivors to manage it by concealment and social withdrawal. However, the understanding of these mechanisms is still theoretical. Most of the hypotheses come from other studies investigating, for example, help-seeking and stigma among those with mental health disorders. But little is known regarding help-seeking behaviors of people bereaved by suicide (Drapeau et al., 2015). Some results from previous research could as well contradict formerly mentioned ideas as the bereaved who go to therapies could also be those who feel more guilt or stigmatization. Studies show that those bereaved who receive sufficient professional support report less feelings of guilt (Schneider et al., 2011). What is more, professional help can help to reduce tension related to stigma (Miller and Kaiser, 2001).

Taking this into account, we find it important to investigate the role of the aspects related to suicide bereavement to seeking help from professionals in the context of other potentially relevant factors. More knowledge in this field would ensure that scientifically based practical recommendations that best fit the needs and interests of the bereaved could be provided. Furthermore, such studies are especially relevant in Lithuania that has one of the highest suicide rates, underdeveloped postvention resources (Klimaite et al., 2017) and extremely little scientific research in this field (Andriessen, 2014).

We raise the hypothesis that those suicide-bereaved who (1) feel less stigmatized, ashamed, guilty after the loss, (2) have lower scores of well-being, (3) are more inclined to reveal distressing information to others and (4) have more positive attitudes toward specialists, are more prone to seek help from mental health professionals. Also we want to investigate whether the mentioned constructs have significant value in predicting if a person had been seeking professional psychological help after the suicide. In addition, we aimed to reveal which barriers to seeking help from professionals the participants identify themselves.

## Materials and Methods

### Participants and Procedures

The sample consisted of 82 adults bereaved by suicide: 64 females (78%) and 18 males, mean age 37.79 years (*SD* = 14.33; range = 19–70 years). 56 (68%) participants lived in a big city, 14 (17%) – in a city, 12 (15%) – in a town/village. Distribution of participants by education: 23 (28%) – primary/secondary, 18 (22%) – vocational, 41 (50%) – university degree; marital status: 14 (17%) – single, 45 (55%) – married/in a long term relationship, 5 (6%) – divorced, 17 (21%) – widow/widower, 1 (1%) – other.

The average time since suicide was 12.23 months (*SD* = 6.52; range from 5 to 36 months). Distribution of participants by the type of relation with the deceased: 55 (67%) lost a member of the nuclear family (10 – father/mother, 4 – sibling, 12 – spouse, 6 – child), 22 (27%) lost another relative, 5 (6%) lost a friend or acquaintance.

Participants were divided into two subsamples by usage of professional psychological help after suicide. It was considered that a participant used professional psychological help if he or she had at least one consultation with a psychologist/psychotherapist, psychiatrist or participated in a support group led by a professional psychologist. No other information regarding help received was required (e.g., the number of therapy sessions). The first subsample consisted of 38 (46%) persons who reported seeking professional help after the suicide.

Participants were recruited nationally via associations of the bereaved by suicide, social media websites, e-mail groups, mental health professionals and snowball sampling. To ensure higher response rates participants could choose between filling in online survey or getting a printed copy by mail. All the participants were contacted by a member of the research team (by e-mail or phone) who explained the aim of the study, participation conditions (voluntary basis, confidentiality, prerogative to refuse participation at any time) and agreed on participation type (online or paper). Then the participants were provided a link to the self-administered survey site or sent a paper questionnaire by mail, which both contained the study consent form. Research team was prepared to provide participants with information about professional help contacts if needed. Up to two reminder e-mails were sent or phone calls were made if participants failed to respond. 18% of the participants who were initially contacted by the research team did not fill in or return the questionnaires. In total, 22 (27%) paper and 60 online questionnaires were filled in.

### Measures

Grief Experience Questionnaire (GEQ) (Barrett and Scott, 1989) is a self-administered instrument measuring various components of grief, including those that have been more associated with grief after suicide. From the original GEQ scale Bailley et al. (2000) found it consists of eight factors. For the purposes of the study we used Stigmatization (10 items), Shame (seven items) and Guilt (six items) subscales of the GEQ. Responses to items in each subscale have a Likert scale from 1 = “never” to 5 = “almost always.” Total scores range from 10 to 50 for the Stigmatization subscale, from 6 to 30 for the Guilt and from 7 to 35 for the Shame subscales, with higher scores indicating higher levels of the perceived stigma, guilt or shame. The reliability evaluations in Cronbach’s alphas for the Stigmatization, Guilt and Shame subscales in our sample were: 0.88,0.87, and 0.81, respectively.

The World Health Organization Well-Being Index (WHO-5) is a 5-item Likert-type scale (from 0 = “at no time” to 5 = “all of the time”) that assesses various aspects (mood, activity, etc.) of psychological well-being during the previous 2 weeks (Psychiatric Research Unit, and WHO Collaborating Centre in Mental Health, 1999). Total score of the scale varies from 0 to 100, where a percentage score of 0 represents worst possible, whereas a score of 100 represents best possible quality of life. The Cronbach’s alpha of WHO-5 in this study was 0.90.

The Distress Disclosure Index (DDI) (Kahn and Hessling, 2001). The 12-item DDI measures the degree to which a person discloses (vs. conceals) emotionally distressing information. Participants respond to items on a five-point scale ranging from 1 = “strongly disagree” to 5 = “strongly agree.” Higher scores indicate a higher willingness to disclose personal information to others. Among the present data, coefficient alpha for scores from the DDI was 0.91.

The Attitudes Toward Seeking Professional Psychological Help – Short Form scale (ATSPPH-SF) (Fischer and Farina, 1995) assesses help-seeking attitudes of the respondents. It consists of 10 items on a four-point Likert-type scale (from 1 = “never” to 5 = “almost always”). Total score ranges from zero to 30, with higher score indicating more favorable attitudes toward seeking professional help. The Cronbach’s alpha of total ATSPPH in this study was 0.83.

The participants were also asked an open-ended question what professional help-seeking barriers they had encountered. Questions measuring demographic, loss-related and help-seeking variables were included in the analysis too.

### Data Analysis

Data were analyzed using SPSS 25.0. Categorical data are reported as percentages, and continuous variables are reported as means and their standard deviations. Pearson correlation analysis was used to investigate the relationship between variables. The comparison analyses of the study groups were carried out using MANOVA. A binary logistic regression analysis was used to evaluate the impact of chosen factors on the likelihood that respondents would report that they contacted a mental health professional after suicide of a loved one. A *p*-value < 0.05 was used as criterion for statistical significance evaluation.

An open-ended question was analyzed by performing thematic analysis based on the steps singled out by Braun and Clarke (2006). The double-coding procedure was chosen to ensure the reliability in this study. If coding discrepancies were found, they were discussed with the help of additional experts in suicidology, final decisions were made.

## Results

There were no statistical differences between the two groups of bereaved people who sought and those who did not seek professional psychological help by participant gender [help-seeking group = 87% female, non-seeking group = 70% female; χ^2^(1,82) = 2.31, *p* = 0.128]; age [help-seeking: *M* = 36.50, *SD* = 12.99, non-seeking: *M* = 38.91, *SD* = 15.46; *t*(80) = 0.76, *p* = 0.451]; education [χ^2^(2,82) = 0.05, *p* = 0.977]; place of residence [χ^2^(2,82) = 1.47, *p* = 0.479]; previous professional psychological help experience [help-seeking group = 41% had previous experience, non-seeking group = 30%, χ^2^(1,81) = 0.64, *p* = 0.423]; time since suicide (help-seeking: *M* = 11.93 months, *SD* = 5.28, non-seeking: *M* = 12.49, *SD* = 7.47; *Z* = −0.30, *p* = 0.762) and type of the relationship with the deceased (help-seeking group = 71% first-degree relative, 29% second-degree relative, friend and others, non-seeking group = 64% first-degree relative, 36% second-degree relative, friend and others) [χ^2^(1,82) = 1.42, *p* = 0.491]. Such results indicate that described characteristics were not significantly important in predicting help-seeking behaviors in this sample.

Among those participants who sought professional help, 32 (84%) consulted psychologist or psychotherapist, 18 (47%) – a psychiatrist and 13 (34%) participated in a support group lead by a professional. 21 (55%) of the participants used more than one kind of professional help for mental health problems due to suicide. Respondents who approached professionals indicated that it was partly to highly helpful as measured on a 1 (“harmful”) to 4 (“very helpful”) (*M* = 3.47, *SD* = 0.63) scale.

Results indicated that stigmatization was positively associated with guilt and shame, and negatively related to well-being. As well as, more positive attitudes toward help-seeking were positively associated with a tendency to disclose oneself (Table 1).

**TABLE 1:** Relationship between the study variables.

	**1**	**2**	**3**	**4**	**5**	**6**
(1) Stigmatization	−					
(2) Shame	0.47***	−				
(3) Guilt	0.44***	0.30**	−			
(4) WHO-5	−0.45***	–0.18	−0.28*	−		
(5) DDI	0.07	–0.12	0.12	0.06	−	
(6) ATSPPH-SF	0.21	0.12	0.26*	0.07	0.39***	−

WHO-5, Well-Being Index; DDI, Distress Disclosure Index; ATSPPH-SF, Attitudes Toward Seeking Professional Psychological Help – Short Form Scale. *p < 0.05, **p < 0.01, ***p < 0.001.

A MANOVA was conducted comparing levels of stigmatization, shame, guilt, well-being, self-disclosure and attitudes toward help-seeking for groups of help-seekers and non-help-seekers. We performed Wilks’ Lambda test which showed significant results, Wilk’s Λ = 0.735 *F*(6,75) = 4.50, *p* = 0.001, $\eta_{\text{p}}^{2}$ = 0.27. Moreover, the results demonstrated significant differences between groups: help-seeking group experienced higher levels of stigmatization and guilt, as well as had more positive attitudes toward seeking help (Table 2).

**TABLE 2:** Results of comparing the scores of the variables by help-seeking behaviors of people bereaved by suicide.

**Suicide bereavement group**	**Help-seeking (*n* = 38)**	**Non-help-seeking (*n* = 44)**			
				
**Outcome measures**	**Mean score (*SD*)**		***F***	***p***	**$\eta_{\text{p}}^{2}$**
Stigmatization	25.58 (9.23)	20.34 (7.32)	8.20	0.005**	0.09
Shame	17.63 (6.05)	16.16 (6.07)	1.20	0.276	0.02
Guilt	21.18 (5.43)	17.68 (6.24)	7.24	0.009**	0.08
WHO-5	35.68 (20.55)	44.73 (23.66)	3.36	0.071	0.04
DDI	42.13 (7.61)	38.43 (10.89)	3.08	0.083	0.04
ATSPPH-SF	32.82 (4.39)	27.18 (6.99)	18.41	< 0.001***	0.19

WHO-5, Well-Being Index; DDI, Distress Disclosure Index; ATSPPH-SF, Attitudes Toward Seeking Professional Psychological Help – Short Form Scale. **p < 0.01, ***p < 0.001.

Binary logistic regression was performed to assess the impact of factors that were found significant in previous analysis on the likelihood that respondents would report that they sought professional psychological help after the suicide. The model contained three independent variables (stigmatization, guilt and attitudes). It was statistically significant χ^2^(3,82) = 24.67, *p* < 0.001, as a whole explained between 25 and 33% of the variance in help-seeking behaviors, and correctly classified 71% of cases. As shown in Table 3, only the attitudes toward seeking help from professionals had significant predictive value.

**TABLE 3:** Logistic regression predicting likelihood of seeking professional psychological help after the suicide.

**Variable**	***B***	***SE***	**Wald**	**OR**	***p***	**95% CI**
Stigmatization	0.07	0.04	2.71	1.06	0.100	0.99–1.14
Guilt	0.04	0.05	0.75	1.04	0.387	0.95–1.14
ATSPPH-SF	0.16	0.05	10.16	1.17	0.001**	1.06–1.30

ATSPPH-SF, Attitudes Toward Seeking Professional Psychological Help – Short Form Scale. **p < 0.01.

Themes on help-seeking barriers identified by the participants themselves are shown in Table 4. Many of the participants indicated no obstacles. The most frequently mentioned obstacles were related to the gaps in the health care system.

**TABLE 4:** Themes on help-seeking barriers identified by the participants.

**Generalizing theme**	**Sub-themes**
No obstacles indicated (51)	No obstacles (41); no need for help (8); did not seek professional help (2)
Health condition (1)	Shock (1)
Personal beliefs and feelings (11)	Stigma of seeking help (2); disbelief that help can be effective (2); fear of speaking with a stranger about sensitive topics (1); preference to cope by oneself (1); guilt due to the loss (1); other undefined internal barriers (4)
Negative experience (5)	Negative previous experience (2); inappropriate specialist (3)
Disappointment in specialists due to suicide (1)	
Gaps in the health care system (33)	Too much effort needed to get an appointment (1); it takes a long time to get help (3); lack of help sources at the place of residence (4); received only short-term help (1); dissatisfaction with medical treatment (2); need for specialists working in private practice (5); financial difficulties (7); Need for active approach from professionals (10): need for specialists’ active role in offering help (2); lack of information on help available (8)
Other individual barriers (3)	Lack of time (2); other individual obstacles that impede access to help sources (1)
Use of other sources of help (4)	

**The number of times the theme was introduced by the participants is shown in brackets.**

## Discussion

In our hypothesis we claimed that suicide bereaved people who feel less stigmatized, ashamed and guilty after the suicide of a loved one should be more prone to seek help from mental health professionals. Our results did not confirm it. On the contrary, it revealed that those bereaved who sought professional support had higher stigmatization and guilt levels. Although the regression analysis did not show the prognostic value of the constructs, differences between the groups of individuals who were and were not seeking help might indicate that stigma and guilt can even act as factors motivating the decision to seek support from professionals. What could be some potential explanations for such findings?

Some studies show that social support can be one of the factors influencing seeking help from mental health professionals. Maulik et al. (2009) found that increased social support was related to less frequent use of formal mental health services. Therefore, the loss of support after the death can encourage the bereaved to look for other ways of getting it. Also, we know that people develop different coping and support strategies to resist stigmatization (Frost, 2011; Hanschmidt et al., 2016). Seeking help from professionals might be one of them. Mental health specialists can create a safe environment for expressing emotions related to prejudice and stigma (Miller and Kaiser, 2001). Furthermore, search for explanation is common among the suicide bereaved (Lindqvist et al., 2008). It might also serve as endeavor to reduce guilt feelings by finding other convincing reasons why the loved one died by suicide (Klimaitė, 2015). For such purposes the bereaved might be hoping for psychoeducation about suicidal processes from mental health professionals as it is noted that information about suicidal and grieving processes facilitate mourning (Klimaitė, 2015). Schneider et al. (2011) found that those bereaved who received sufficient professional support reported less feelings of guilt. So those who feel more guilt about the suicide might have more reasons to take advantage of professionals in reducing those unpleasant feelings.

Another possible explanation is the impact of stigmatization and guilt to the health outcomes of the bereaved. As we discussed earlier, it is found that stigma is related to mourning difficulties (Feigelman et al., 2009), intense guilt feelings can have negative psychological consequences too (Clark, 2012). In our study stigmatization and guilt were negatively associated with well-being of the participants. This means that people who feel more stigmatization or guilt also can have more complicated grief and intense unpleasant mourning reactions. Testing hypotheses about indirect effects of stigmatization and guilt to help-seeking would require future research.

The study revealed that the only significant factor in predicting whether a person sought help after the suicide was attitudes toward seeking professional psychological care. The role of attitudes toward seeking help was found to be important in previous research too (Picco et al., 2016). The results confirm the significance of programs targeted at changing attitudes toward seeking help from mental health professionals.

We did not find statistical differences of shame levels between the groups. According to some authors, external shame (in this case, perceived stigmatization) is more important to humans than the internal one because of our evolutionary based need for belonging to a group (Kim et al., 2011). It would explain our results. However, as shame can be seen as internalized stigma (Hanschmidt et al., 2016) we hypothesize that the relation between professional help-seeking and shame might be more complicated and more difficult to capture.

Adaptation to the changes after the loss was evaluated by analyzing the index of well-being. No significant differences were found between the subjective well-being (WHO-5) of the bereaved who sought professional help and those participants who did not seek it. Of course, the survivors had already received or had been receiving help before the moment of participation in the study. So it is likely that those bereaved who got treatment were less depressed than before the intervention. However, the results did not reveal that health condition act as a help-seeking barrier. It was mentioned by the participants themselves only once too.

We also found that participants who contacted specialists were more inclined to disclose distress to others, but the difference was not significant. Although some studies show that a tendency to reveal emotionally relevant information to others is related to the attitudes or intentions of seeking help (Nam et al., 2013), it can be that in the face of severe emotional stress even those people who are not open about their emotional problems in their everyday life seek support from professionals. We also did not find significant differences between the groups regarding receiving professional psychological help before the death. These results also confirm that the bereaved sought new ways of help resources independently from their previous experiences.

The participants themselves highlighted the gaps in the health care system as main barriers to seeking help from mental health professionals. A need for active approach from professionals and lack of information on help available were mentioned the most often. The participants noted financial difficulties as barriers and a need for specialists working in private practice. The idea that the participants did not see seeking help from public mental health specialists (that is free of charge in Lithuania) as an option might imply mistrust in public mental health care system. Individual factors were mentioned less often. Such results reflect the importance of improving health care system to ensure proper and easily approachable help resources. However, it is also a reminder that the position of “I do not need help” might mask other important individual factors that cannot be easily captured by qualitative methods.

One of the limitations of our study was that participation percentage of men was much lower. Although, there were no differences in our sample, some studies find that help-seeking behaviors of men and women might be variant (Vogel et al., 2007). However, male non-response bias is very often in suicide bereavement research (Pitman et al., 2016). Also, the inclusion of the participants into our sample was not random. High participation avoidance was noticed in previous suicide bereavement studies in Lithuania too (Klimaitė, 2015) so investigating help-seeking behaviors of people who do not seek or are even against mental health professionals remains a challenge. Another limitation is that in our study we did not differentiate between seeking help from distinct professionals (ex. psychologists and psychiatrists) and did not have more detailed information about help received. Although, some studies show that attitudes toward mental health specialists are shared at least to some degree (Barney et al., 2006), it would be important to apply more nuanced measures of help-seeking process than we were able to use in our broad-based survey. Furthermore, in our study we had retrospective evaluations of respondents’ previous experiences. As it was not a longitudinal study design we did not have a chance to capture changes in measured constructs before and after the professional intervention.

Vogel et al. (2007) notice that help-seeking does not constitute of a single act or choice, it is a process that often encompasses conflicts between various approach and avoidance factors, so it is not easy to evaluate general tendencies of peoples’ help-seeking behaviors. As one bereaved by suicide illustrated the complexity of the phenomena by identifying guilt as a barrier (“The hardest thing was to get over myself, accusations of myself appeared to be very reasonable at first”) as well as the reduction of it as one of the main advantage (“My psychologist introduced me with the process of bereavement. I especially needed to become aware of the fact that this was not my responsibility”) of help-seeking (Geležėlytė, 2019).

It is also worth mentioning that cultural context is important in studies investigating such culturally sensitive phenomena as guilt, shame, stigmatization and grief itself (Li et al., 2014; Pitman et al., 2016). Lithuania is among countries with highest suicide mortality that might have culturally ingrained reasons too (Gailienė, 2015). Authors also claim that there are specific reasons for stigmatization of suicide in the country due to the history of occupations and cultural trauma (Klimaite et al., 2017). Having in mind that comparison of help-seeking behaviors of suicide bereaved people is novel, it is crucial to continue such studies and compare the results in different cultures.

## Conclusion

Despite limitations, the results of the study challenge previously spread notion that stigmatization, guilt and shame after suicide of the loved one can act only as help-seeking barriers. The findings indicate that those bereaved who feel more stigma and guilt might contact professionals more often. Also the results showed the attitudes toward mental health specialists had the highest prognostic value in predicting help-seeking behaviors of the bereaved. The participants themselves identified the gaps in the health care system as main barriers to seeking help.

## Data Availability Statement

The datasets generated for this study are available on request to the corresponding author.

## Ethics Statement

Approval for the current study was obtained from the Psychology Research Ethics Committee of Vilnius University. The participants provided their informed consent to participate in this study.

## Author Contributions

OG and DaG designed and carried out the study. SD and DoG collected the data and contributed to the writing of the manuscript. OG, JL, and EM-R performed the initial analyses, and contributed to the writing of the manuscript. DaG and PS assisted in the writing of the manuscript and supervised the study. All authors revised and approved the submitted version.

## Conflict of Interest

The authors declare that the research was conducted in the absence of any commercial or financial relationships that could be construed as a potential conflict of interest.
